# A new biotechnology for *in-planta* gene editing and its application in promoting flavonoid biosynthesis in bamboo leaves

**DOI:** 10.1186/s13007-023-00993-4

**Published:** 2023-03-02

**Authors:** Huayu Sun, Sining Wang, Chenglei Zhu, Kebin Yang, Yan Liu, Zhimin Gao

**Affiliations:** 1 Key Laboratory of National Forestry and Grassland Administration/Beijing for Bamboo & Rattan Science and Technology, Beijing, 100102 China; 2 Institute of Gene Science and Industrialization for Bamboo and Rattan Resources, International Centre for Bamboo and Rattan, Beijing, 100102 China

**Keywords:** Bamboo, *In-planta* transformation, Gene editing, Violaxanthin de-epoxidase, Bamboo leaf flavonoid

## Abstract

**Background:**

Bamboo is a perennial and renewable biomass forest resource and its leaf flavonoid is an antioxidant for biological and pharmacological research. The established genetic transformation and gene editing systems in bamboo are significantly limited by the dependence on bamboo regeneration capability. The way to improve the flavonoid content in bamboo leaves through biotechnology is still not feasible.

**Results:**

Here, we developed an *in-planta*, *Agrobacterium*-mediated gene expression method for exogenous genes via wounding and vacuum in bamboo. We demonstrated that the *RUBY* served as a reporter efficiently expressed in bamboo leaves and shoots, albeit unable to integrate into the chromosome. We have also developed a gene editing system by creating an in situ mutant of the bamboo violaxanthin de-epoxidase (*PeVDE*) gene in bamboo leaves, with lower NPQ values under the fluorometer, which can serve as a native reporter for gene editing. Furthermore, the bamboo leaves with increased flavonoid content were achieved by knocking out the cinnamoyl-CoA reductase genes.

**Conclusions:**

Our method can be applied for the functional characterization of novel genes in a short time and is helpful for bamboo leaf flavonoid biotechnology breeding in the future.

**Supplementary Information:**

The online version contains supplementary material available at 10.1186/s13007-023-00993-4.

## Background

Bamboo is an evergreen and renewable forest biomass resource, which is not only widely used in manufacturing timber, artwork, and papermaking, but also in biological and pharmacological research. Bamboo leaf flavonoids have a variety of activities such as antioxidant, anti-inflammatory, anti-ulcer, and antitumor, which can help reduce the risk of cardiovascular disease, Alzheimer’s disease, Parkinson’s disease, and diabetes in previous studies [1, 2]. Genetic transformation and gene editing systems in bamboo have been established via callus induction [3–5]. However, the application of the existing gene editing systems in bamboo is primarily restricted by the dependency on the regeneration capability of bamboo, which is highly time-consuming (lasting usually over one year), labor-intensive, and complicated. Bamboo has an unpredictable and very long flowering cycle, usually lasting in vegetative growth for a few decades before flowering, which makes it difficult to obtain suitable explant materials, including flowers and immature embryos for callus induction. Therefore, an easy and feasible method for rapid gene expression and gene editing is urgently necessary for bamboo research.

The *Agrobacterium*-mediated transient expression can be easily conducted without using expensive equipment or complicated procedures compared with other transient expression methods, such as biolistic bombardment, PEG-mediated gene transfer, and protoplast electroporation. After being infected by *Agro*-infiltration or *Agro*-injection, T-DNA will be transferred from the bacteria to plant cells. All pieces of foreign DNA are transcriptionally competent, only a small percentage of the T-DNA can be integrated into the host chromosome, leading to stably transformed cells that can subsequently be regenerated into transgenic plants [6]. Moreover, a previous study found that the non-integrated transient CRISPR/Cas9 expression can obtain mutation in tobacco [7]. Although *Agrobacterium*-mediated transient gene expression can be easily and widely performed in dicotyledons, the strategies for the transformation of monocots are relatively inefficient [8]. Therefore, transient gene expression and gene editing are challenging in monocots, especially bamboo.

Here, we describe an *in-planta*, *Agrobacterium*-mediated transient gene expression strategy for exogenous genes in bamboo. We have also developed a gene editing system by creating an in situ mutant of the bamboo violaxanthin de-epoxidase (*PeVDE*) gene in bamboo leaves, with lower values of non-photochemical quenching (NPQ). We further investigate that the edited *PeVDE* mutant can serve as a stable reporter for gene expression and gene editing without selection pressure and that the mutant of other genes can be easily detected by chlorophyll fluorescence imaging. To apply this technology in bamboo, we knock out cinnamoyl-CoA reductase (CCR) genes by CRISPR/Cas9 and generated the mutants with the increase of flavonoid content in bamboo leaves. Our method will be applied for the functional characterization of novel genes and *in-planta* gene editing in bamboo and other plants.

## Methods

### Plant materials and growth conditions

The calli induced from immature embryos of moso bamboo (*P. edulis*) were kindly provided by Ms. Guirong Qiao from the Chinese Academy of Forestry, Hangzhou, China. Moso bamboo seedlings were generated from seeds. After being soaked in water for two days, the seeds were sowed into a substrate with a mixture of soil and vermiculite (3:1) for germination and seedling growth under laboratory conditions at 18–25 °C. A 16 h light/8 h dark photoperiod, and the intensity of light in the light phase was 250–350 μmol m^−2^ s^−1^. The half-month-old seedlings with a height of 2–10 cm were used for *Agro-*infection.

*P. aureosulcata* f. *spectabilis* (Jinxiangyuzhu) and *P. aureosulcata* f. *aureocarlis* (Huangganjingzhu) were planted in the garden of the International Center for Bamboo and Rattan, in Beijing, China. The emerging bamboo shoots with a height of 5–20 cm were used for *Agro-*infection.

### Plasmids and transformation.

The pHDE-*35S*::*RUBY* construct carrying the *RUBY* reporter gene under the control of the *CaMV 35S* promoter (*35S*::*RUBY*) was purchased from Addgene (Catalog number: 160908). The pC1300-*Ubi*::*Cas9* [9] plasmid was kindly provided by Ms. Guirong Qiao.

The sgRNA guiding sequences of *PeVDE* and *PeCCR*s were inserted into the pC1300-*Ubi*::*Cas9* vector, between the two *Aar*I sites using annealed oligonucleotides (Additional file 3: Table S2).

The plasmids were individually transformed into the AGL1, GV3101, LBA4044, and EHA105 strains of *A. tumefaciens*. *Agrobacterium* in YEP medium with the antibiotics was grown at 28 °C to an OD_600_ of 0.8. Suspensions of *Agrobacterium* were resuspended in the infiltration medium [10 mM MgCl_2_ and 10 mM MES-KOH (pH 5.6)] to an OD_600_ of 0.6.

### *Agrobacterium*-mediated* in-planta* transformation system

**Fig. 1:**
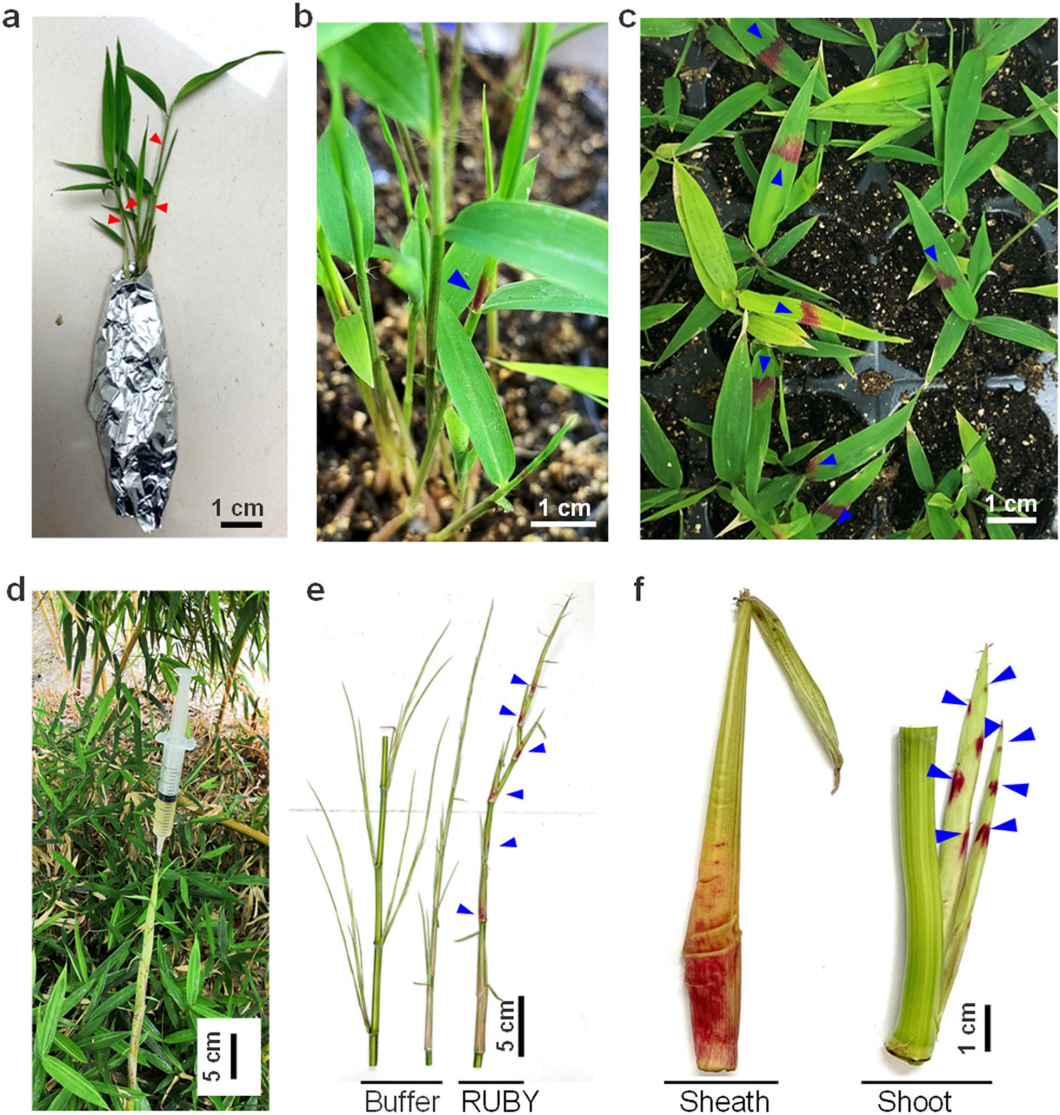
Expression of *RUBY* and accumulation of betalain in bamboo leaves and young shoots. **a** Bamboo (*Phyllostachys edulis*) seedlings ready for infection with *Agrobacterium*. The red triangles indicate the positions of the wounds. **b**, **c** Phenotype of *P. edulis* leaves showing betalain accumulation after 3 days (**b**) and 5 days (**c**) of infection. **d** Bamboo (*P. aureosulcata* f. *spectabilis*) shoots in the fields, ready for infection with *Agrobacterium* by injection. **e**, **f** Young shoots showing betalain accumulation. The blue triangles represent the areas with betalain accumulation

Transformation of bamboo seedlings: The seedlings were transferred from the normal culture conditions to that of higher humidity (RH > 90%) and lower illumination intensity (less than 50 μmol m^−2^ s^−1^) for 2 h. Prior to infiltration, the top part of bamboo seedlings were wound by a sharp needle (the positions were marked by red triangles in Fig. 1a). Then, the upper part of the seedlings were immediately immersed into suspensions of *Agrobacterium* and immediately transferred to vacuum conditions of 25–27 inches of Hg for 2 min. After infiltration, the treated seedlings were immediately placed in dim light or dark conditions (less than 50 μmol m^−2^ s^−1^) at room temperature (18–25 °C) with high humidity (RH > 90%) for 1–2 days.

Transformation of bamboo shoots under field conditions: A syringe was used for injecting the *Agrobacterium* suspension into the shoot tips. The syringe needle was used to pierce into the shoot tips directly without stripping the sheaths. The depth of the injection was adjusted to ensure that the suspension reached the hollow pith and the space inside the sheaths.

### Primer design, PCR, and sequencing

The specific primers for amplification of the *PeVDE*, *PeCCR*s, and *RUBY* fragments were designed manually. All the primer sequences used for gene cloning and PCR analyses were listed in the Additional file 3: Table S2. High-sensitive Polymerase Chain Reaction (PCR) was performed using the PrimeSTAR Max DNA polymerase (Takara, Japan), which has high fidelity and amplification efficiency in gene cloning. The direct PCR products were sent to TsingkeBiotechnology (Beijing, China) for deep sequencing.

### Measurement of NPQ values

The in vivo PSII chlorophyll fluorescence of bamboo leaves was measured with the actinic light of an IMAGING-PAM fluorometer (Walz, Effeltrich). Prior to measurements, the bamboo seedlings were placed under conditions of high light intensity (1200 μmol m^−2^ s^−1^) for 2 h. The intensity of the actinic light was set to 800 μmol m^–2^ s^–1^. The NPQ was calculated using the formula: NPQ = (*F*_m_—*F*_m_’)/*F*_m_’, where *F*_m_ represented the maximum fluorescence in the dark-adapted state, and *F*_m_’ represented the maximum fluorescence in any light-adapted state [10]. The values of NPQ were monitored from the visual interface of the ImagingWin software (Walz, Effeltrich, Germany).

### Measurement of flavonoid content

The total bamboo leaf flavonoid content was determined by aluminum chloride assay [11, 12].

### Measurement of lignin content

The lignin content was determined by a lignin assay kit (Solarbio, China) according to the protocol provided by the manufacturer.

## Results

### Rapid and transient *Agrobacterium*-mediated *in-planta* gene expression in bamboo leaves and shoots

RUBY is a reporter with the function of converting tyrosine to vividly red betalain [13], which accumulated products can be visualized with the naked eye and will be a suitable reporter in transient gene expression system. We conducted moso bamboo (*Phyllostachys edulis*) callus transformation with *RUBY* (*35S*::*RUBY*) and *GUS* (*35S*::*GUS*) reporter genes by Huang et al. (2022) method, respectively. In our trial, the transformation completely didn’t work in the subcultured calli (> 12-month-old calli). Meanwhile, the method for dicot *Agro*-infiltration was also completely inefficient on bamboo seedlings.

Next, we developed an *in*-*planta Agrobacterium*-mediated method for the transient expression of the exogenous *RUBY* gene in the leaves and shoots of bamboo. The red color of the immature leaves was visible to the naked eye after three days of infection and became more vivid after the rolled leaves unfolded (blue triangle, Fig. 1b, c). The findings revealed that the exogenous *RUBY* gene was expressed and that betalain synthesis occurred in the immature leaves of the bamboo seedlings.

The percentage of bamboo seedlings that accumulated betalain after being infected by *Agrobacterium* GV3101 strain was the highest, with 85.20% in younger and 91.70% in elder seedlings respectively (Additional file 2: Table S1). The results also demonstrated that the *Agrobacterium* strain of GV3101 induced less physical injury than AGL1, EHA105, and LBA4404 (Additional file 1: Fig. S1). Furthermore, suspensions of *Agrobacterium* were injected with positive pressure into bamboo shoots harboring the *RUBY* gene under field conditions (Fig. 1d). As the shoots grew, betalain accumulation was evident in both the shoot sheaths and young lateral shoots after 15 days of infection (blue triangle, Fig. 1e, f; Additional file 1: Fig. S2 and S3).

High-sensitive PCR was performed using the template DNA extracted from the leaves showing betalain accumulation for determining the integration of the T-DNA into the bamboo genome. After 40 cycles of PCR, fragments of the *RUBY* gene were still not detected in the genomic DNA of bamboo leaves showing betalain accumulation after 30 days of infection (Additional file 1: Fig. S4), indicating that the host cell had either rejected the inserted T-DNA, or the number of integrated fragments were too low to be detected. We also observed that almost all the red betalain color will disappear in bamboo leaves after three months of infection. Therefore, the reporter gene, such as *RUBY*, can not stably exist in the non-integrated transient expression system.

### *In-planta* gene editing of bamboo violaxanthin de-epoxidase gene (*PeVDE*)

**Fig. 2:**
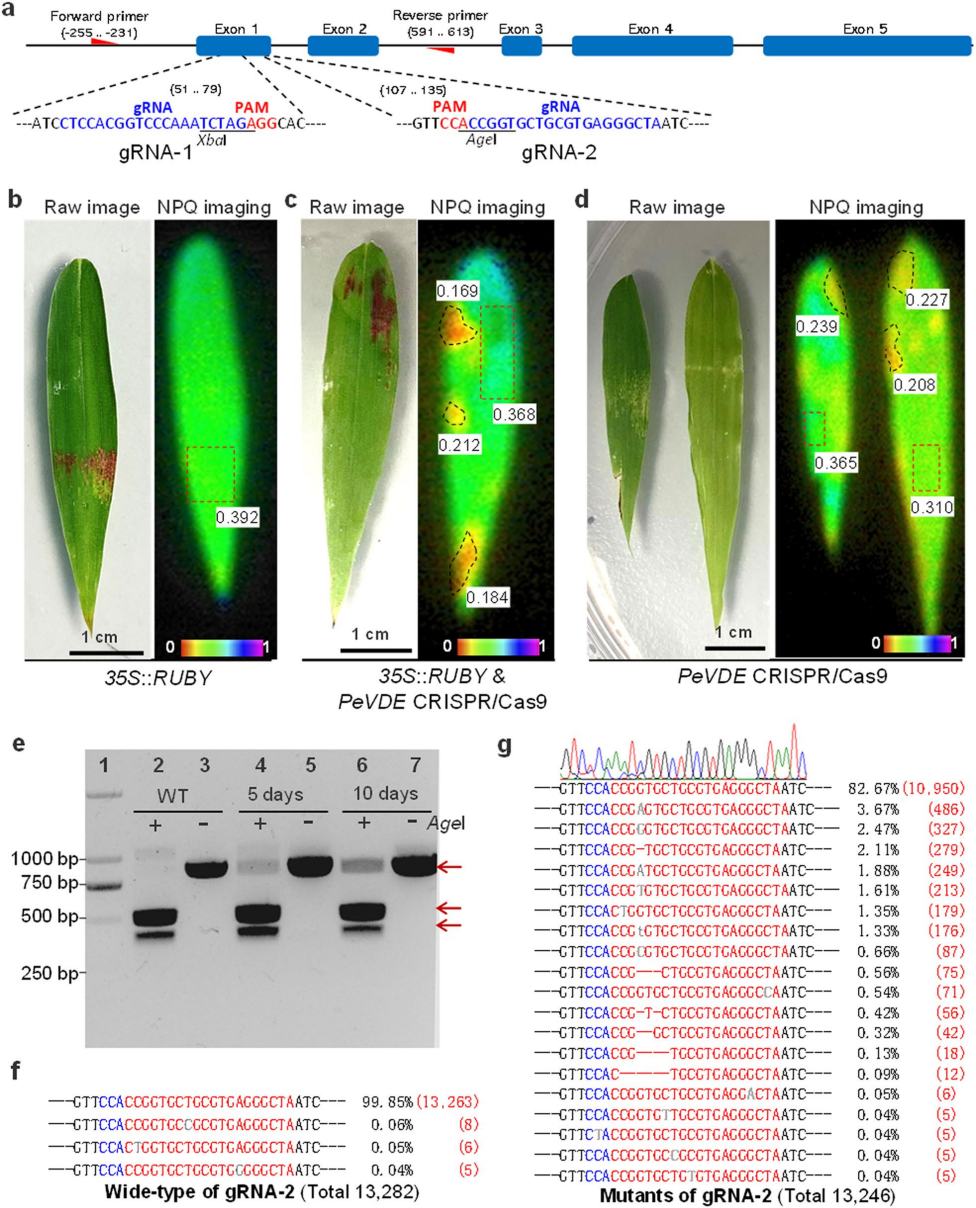
*In-planta* gene editing of *PeVDE* gene in bamboo using CRISPR/Cas9 technology. **a** Structure of the *PeVDE* gene and sequences of the target sites. The red triangles represent the forward and reverse primers. **b**–**d** NPQ imaging of leaves following infection with *Agrobacterium* harboring different constructs: *35S*::*RUBY* (**b**), the mixture of *35S*::*RUBY* and *PeVDE* CRISPR/Cas9 (**c**), *PeVDE* CRISPR/Cas9 (**d**). The numbers represent the NPQ values of the infected and non-infected areas. **e** Results of PCR of genomic DNA obtained from wild-type (WT) non-infected and infected leaves using gRNA-2 after 5 or 10 days of infection. + and—represent PCR products with or without *Age*I digestion, the different bands are indicated with red arrows. **f**, **g** Deep sequencing results of the wide-type (**f**) and mutated *PeVDE* clones edited using gRNA-2 (**g**). Portions of sequences in red, blue, and grey indicate the target sites, PAM, and insertions, respectively. The red dashes indicate deleted nucleotides

Then, in order to investigate whether the transient expression of CRISPR/Cas9 could serve as an efficient gene editing system in bamboo, we selected the bamboo violaxanthin de-epoxidase gene (*PeVDE*), which is responsible for the synthesis of zeaxanthin to dissipate the excess absorbed light energy as thermal energy [14, 15], for trial gene editing studies. Two guide RNA (gRNA) target sites were designed in the first exon of *PeVDE* at the restriction sites of *Xba*I (gRNA-1) and *Age*I (gRNA-2), upstream of the protospacer adjacent motif (PAM) (Fig. 2a). These restriction sites at the target areas might be lost due to mutations. Firstly, the *Agrobacterium* harboring *PeVDE* CRISPR/Cas9 was used in the transient expression by co-infiltrated with the *Agrobacterium* containing *RUBY* reporter with the ratio of 1:3. Compared to the infiltration only with RUBY reporter (Fig. 2b), we observed that certain areas in the leaf blades had lower non-photochemical quenching (NPQ) values after co-infiltration for five days (Fig. 2c). Due to the betalain accumulation hardly affecting the NPQ values of leaves (Fig. 2b), the areas with lower and higher NPQ values were separated and used for DNA extraction, respectively. The mutant of *PeVDE* sequences in those areas with lower NPQ values (Fig. 2d) was confirmed by digestion with the aforementioned restriction enzymes (Fig. 2e; Additional file 1: Fig. S5a), which indicated that the *PeVDE* was edited by the transient gene editing system. Meanwhile, the areas with lower NPQ values were used for DNA extraction and deep sequencing (Fig. 2f, g; Additional file 1: Fig. S5b, S5c). The results of sequencing further confirmed that the *PeVDE* was edited by the transient gene editing system and the mutation rate of gRNA-2 was 17.33% (2,296 mutant sequences in 13,246) (Fig. 2g). Similar results were obtained for gRNA-1 with an edited efficiency of 10.6% (1286 mutant sequences in 12,142), which was relatively lower than that of gRNA-2 (Additional file 1: Fig. S5). The results indicated that the *PeVDE* mutant decreased the leaf dissipation capacity of excess absorbed light energy, which could be clearly monitored by a chlorophyll fluorometer.

### *In-planta* gene editing of bamboo cinnamoyl-CoA reductase genes with the edited *PeVDE* mutant as a reporter

**Fig. 3:**
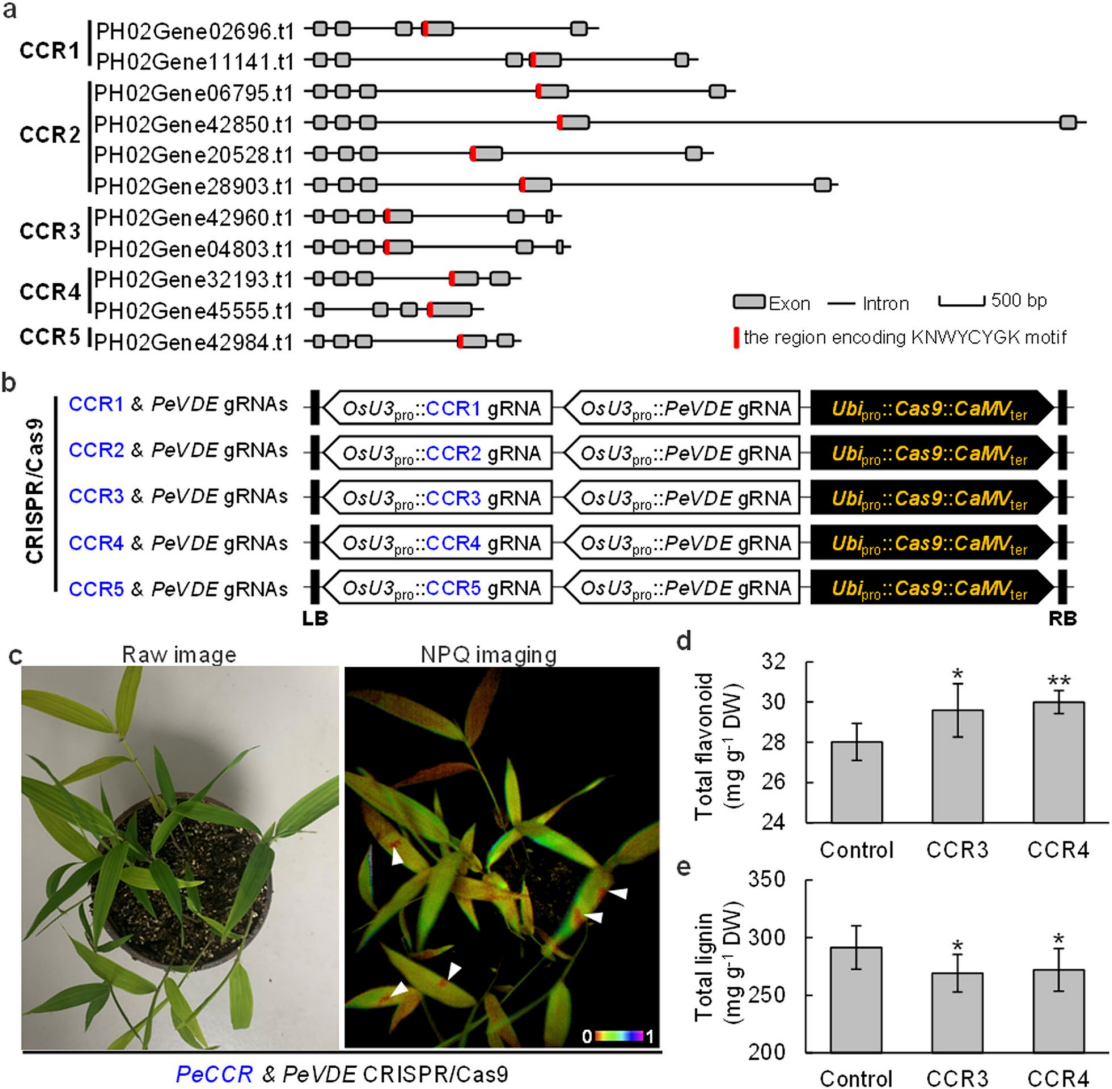
Edited *PeVDE* mutant served as a reporter for *in-planta* gene editing of *PeCCR*s. **a** Gene structures of *PeCCR*s and the region in genome encoding the conserved motif of KNWYCYGK. **b** The CRISPR/Cas9 constructs carrying the gRNAs of *PeVDE* and *PeCCR*s. **c** Images of leaves after infection with *Agrobacterium* harboring the constructs. White triangles represent areas with lower NPQ values. **d**, **e** Total flavonoid (**d**) and lignin (**e**) content in the infected leaves with lower NPQ values. * and ** indicate significant differences at *p* ≤ 0.05 and *p* ≤ 0.01 by ANOVA, respectively. Data are means ± AVEDEV (n = 3). Exact *p* values from CCR3 and CCR4 are as follows: **d** 0.0242 and 0.0069, **e** 0.0264 and 0.026

Furthermore, we applied the edited *PeVDE* mutant as a reporter in the non-integrated transient gene expression system. Cinnamoyl-CoA reductase (CCR) catalyzes the reduction reaction to synthesize *p*-coumaraldehyde, caffealdehyde, coniferaldehyde, 5H coniferaldehyde, and sinapaldehyde in the monolignol pathway for lignin biosynthesis [16]. Inhibiting CCR could promote flavonoid biosynthesis and reduce lignin production; opposite results were observed under the background of overexpressed *CCR* in apple [17]. Here, all the CCR genes in moso bamboo were genome-wide identified and the gRNA target sites of 11 *PeCCR*s were designed according to their sequences encoding the conserved motif of KNWYCYGK, which was critical for the catalysis of CCRs [18] (Fig. 3a; Additional file 3: Table S2). Five gRNAs were designed to target the five groups respectively. Specifically, CCR1 gRNA targeted the genes of PH02Gene02696.t1 and PH02Gene11141.t1; CCR2 gRNA targeted the genes of PH02Gene06795.t1, PH02Gene42850.t1, PH02Gene20528.t1, and PH02Gene28903.t1; CCR3 gRNA targeted the genes of PH02Gene42960.t1 and PH02Gene04803.t1; CCR4 gRNA targeted the gene of PH02Gene32193.t1 and PH02Gene45555.t1; CCR5 gRNA targeted the gene of PH02Gene42984.t1 (Additional file 3: Table S2). Then, the CRISPR/Cas9 vector carrying the gRNAs of *PeVDE* and *PeCCR*s respectively (Fig. 3b) was transfected into *Agrobacterium* for the transformation of bamboo leaves.

Culture for 30 days after infection, the seedlings were treated with light of high intensity (1200 μmol m^−2^ s^−1^) for 20 min for ensuring the absorption of excess light energy. The results of chlorophyll fluorescence measurement showed that no areas with decreased NPQ values were found in the leaves transfected by the CRISPR/Cas9 vector carrying the gRNAs of *PeCCR*s alone, which indicated that the edited *PeCCR*s had no effect on the NPQ value. Meanwhile, the areas of the leaves (indicated by white triangles) transfected by the CRISPR/Cas9 vector carrying the gRNAs of *PeVDE* and *PeCCR*s had lower NPQ values (right panel, Fig. 3c), which were detached for the validation of mutation and the determination of flavonoid and lignin.

The sequencing results revealed that the *PeCCR*s were edited by the transient gene editing system, and the mutation rates of the gRNAs for CCR1, CCR2, CCR3, CCR4, and CCR5 were 11.4% (138 mutant sequences in 1212) (Additional file 1: Fig. S6), 13.1% (228 mutant sequences in 1747) (Additional file 1: Fig. S7), 9.1% (513 mutant sequences in 5627) (Additional file 1: Fig. S8), 10.0% (331 mutant sequences in 3285) (Additional file 1: Fig. S9), and 9.3% (222 mutant sequences in 2687) (Additional file 1: Fig. S10) respectively. The results also indicated that the *PeVDE* mutant could be used as a reporter to screen the genome editing materials for other genes. Furthermore, we also checked the potential off-target editing by Cas9 in the transient gene editing system. In the CCR4 (PH02Gene32193.t1 and PH02Gene45555.t1) genes, the target site had only one base pair mismatched with the target site in CCR5 (PH02Gene42984.t1) gene, which was also recognized and cleaved by Cas9, generating the mutation rate of 1.4% (64 mutant sequences in 4684) (Additional file 1: Fig. S11). Also, the mutation rate of CCR5 by the gRNA of CCR4 was 1.3% (32 mutant sequences in 2432) (Additional file 1: Fig. S12).

The content measurement of flavonoid and lignin in the edited *PeCCR*s leaf areas showed that the mutants of CCR3 (PH02Gene42960.t1 and PH02Gene04803.t1) and CCR4 (PH02Gene32193.t1 and PH02Gene45555.t1) promoted the biosynthesis of flavonoid (Fig. 3d; Additional file 1: Fig. S13a) and reduced the biosynthesis of lignin (Fig. 3e; Additional file 1: Fig. S13b). In addition, VDE is a highly conserved protein with three conserved domains, namely, Cys-rich, lipocalin, and Glu-rich domains, in plants [14, 19], and the *VDE* mutant can also be used as a reporter in *in-planta* non-transgenic gene editing systems in other plants.

## Discussion

The special biological characteristics of bamboo, such as the unpredictable flowering and long flowering cycle, have always been the main factor limiting the progress of bamboo breeding. In order to speed up the breeding process, people try to use modern biotechnology to carry out a lot of research work, such as space breeding, mutation breeding, and genetic engineering breeding. Recently, genetic transformation and gene editing systems have been reported in bamboo [3–5], and all of them are using callus for transformation through *Agrobacterium*. However, the callus induction and regeneration process is time-consuming, labor-intensive, and complicated. Also, these existing genetic transformation and gene editing systems are primarily restricted by the dependency on the regeneration capabilities of bamboo callus. Meanwhile, it is difficult to obtain suitable immature embryos for callus induction. It has been reported that the age of the callus is an important factor influencing the transformation efficiency [20]. The callus we used for transformation has been subcultured for more than one year, and our results show that the transformation is completely inefficient. Therefore, it is urgent to develop a rapid and efficient method for gene function identification in bamboo. Based on previous literature and experimental research, we successfully develop a time-saving *in-planta* gene expression strategy for exogenous genes in bamboo, without the complicated process of callus induction and regeneration. The *RUBY* served as a reporter that can efficiently express in bamboo leaves and shoots by *Agrobacterium*-mediated and vacuum transformation simultaneously, although the *RUBY* gene is unable to integrate into the chromosome of bamboo. This transient expression method will be widely applied for the functional characterization of novel genes in bamboo in the future.

After *Agro-*infection, the T-DNA fragments will be transferred to plant cells and only a small percentage of the fragments can be integrated into the host chromosome. In most cases, the maximum level of non-integrated transient gene expression occurs at 3–4 days after infiltration and fade rapidly after 5–6 days [8]. Thus, a stable reporter gene in the non-integrated transient gene expression system is an objective that plant biotechnologists pursue. The application of CRISPR/Cas9 always entails the stable integration of Cas9 endonuclease and gRNA genes into its chromosome, the transient expression fragments still can obtain mutation in plants [7]. Although the mutant of the phytoene desaturase (PDS) gene caused by the non-integrated gene editing can stably exist after the degradation of the non-integrated T-DNA fragments [7], the *pds* mutant has the disability of catalyzing the desaturation of colorless phytoene into *z*-carotene and the albinism retards the plant growth [21, 22]. Zeaxanthin is synthesized by violaxanthin de-epoxidase (VDE) and its content is highly correlated with the value of NPQ, which can dissipate the excess absorbed light energy as thermal energy to prevent photoinhibition [14, 15, 19]. We have developed an *in-planta* gene editing system via transient expression of CRISPR/Cas9 in bamboo leaves. The edited *PeVDE* mutant has the phenotype of lower NPQ value and which can be easily detected by chlorophyll fluorometer. Furthermore, gene editing of *PeCCR*s was performed with the edited *PeVDE* as a native reporter, the different mutation rates of CCR1 to CCR5 were confirmed by sequencing, and the increased flavonoid content and decreased lignin content were achieved in the edited bamboo leaves.

In our trials, the transient expression only occurs in the leaves, shoot sheaths, and young lateral shoots, whose tissues cannot generate germline-edited or transgenic progeny in bamboo. While the bamboo species has unique characteristics of a long and unpredictable flowering events, *in-planta* gene editing is not needed to obtain germline-edited or transgenic progeny in bamboo. In our future study, we will focus on the shoot meristem cells where gene editing can take place. Newly formed shoots derived from gene-edited shoot meristem cells will therefore be edited. Such a procedure can be easy and simple to perform because it requires no tissue culture, plant regeneration, or genetic transformation. Thus, our study develops a new reporter for screening the mutant of other genes in the transient gene expression system of bamboo, which can be applied for the functional characterization of novel genes and *in-planta* gene editing in bamboo and other plants.

## Conclusions

In conclusion, GV3101 is an effective *Agrobacterium* strain, which can meditate the expression of *RUBY* used as a reporter for monitoring transient *in-planta* gene expression in bamboo with visibly vivid red coloration. Furthermore, gene editing is successfully conducted in bamboo leaves, the edited *PeVDE* mutant of bamboo can be used as a native reporter gene for screening mutant tissues by gene editing with non-integrated transient gene expression system. The increased flavonoid content is successfully achieved by gene editing of *PeCCR*s with the edited *PeVDE* as a native reporter. The method provides a non-transgenic strategy for *in-planta* gene editing, which can be used as a platform for the functional characterization of endogenous genes.

## Supplementary Information

**Additional file 1: Fig. S1. **The leaf phenotype of *Phyllostachys edulis* seedlings with betalain accumulation. The *Agrobacterium tumefaciens* strains of AGL1, LBA4404, EHA105, and GV3101 harboring *35S*::*RUBY* construct were used to infiltrate by vacuum respectively. The red triangles indicated the positions of the wounds. **Fig. S2.** The shoot phenotype of *Phyllostachys aureosulcata* *Spectabilis*’ with betalain accumulation after infection for 15 days. **a**, Shoots injected with *Agrobacterium*-free suspension. **b**, Shoots injected with the buffer solution with *Agrobacterium* suspension harboring *35S*:*RUBY* construct. Black box, Magnified image. **Fig. S3. **The shoot phenotype of *Phyllostachys aureosulcata* '*Aureocarlis*' with betalain accumulation after infection for 15 days. **a**, Shoots injected with *Agrobacterium*-free suspension. **b**, Shoots injected with the buffer solution with *Agrobacterium* suspension harboring *35S*:*RUBY* construct. Black box, Magnified image. **Fig. S4. ***RUBY* reporter gene detected in the red leaf areas with betalain accumulation using PCR. Lane1, the non-infected leaves as the negative control; Lane2 and 3, the betalain accumulated leaves after *Agrobacterium* infection for three days and 30 days respectively; Lane4, the *35S*::*RUBY* plasmid as positive control. *PeActin *(GenBank Accession No. GU434145) was used as endogenous control under the same PCR conditions. **Fig. S5. **The detection of gene-edited target1 of *PeVDE* in bamboo leaves. **a**, Results of PCR of genomic DNA obtained from wild-type (WT) non-infected and infected leaves using gRNA-1 after infection for 5 or 10 days. + and - represent PCR products with or without *Xba*I digestion. The different bands were indicated with red arrows. **b–c**, Deep sequencing results of wide-type (**b**) and mutated *PeVDE* clones edited using gRNA-1 (**c**). Portions of sequences in red, blue, and grey indicated the target sites, PAM, and insertions, respectively. The red dashes indicated the deleted nucleotides. **Fig. S6. **Deep sequencing results of gene edited of CCR1 in bamboo leaves. Portions of sequences in red, blue, and grey indicated the target sites, PAM, and insertions, respectively. The red dashes indicated the deleted nucleotides. **Fig. S7. **Deep sequencing results of gene edited of CCR2 in bamboo leaves. Portions of sequences in red, blue, and grey indicated the target sites, PAM, and insertions, respectively. The red dashes indicated the deleted nucleotides. **Fig. S8. **Deep sequencing results of gene edited of CCR3 in bamboo leaves. Portions of sequences in red, blue, and grey indicated the target sites, PAM, and insertions, respectively. The red dashes indicated the deleted nucleotides. **Fig. S9. **Deep sequencing results of gene edited of CCR4 in bamboo leaves. Portions of sequences in red, blue, and grey indicated the target sites, PAM, and insertions, respectively. The red dashes indicated the deleted nucleotides. **Fig. S10. **Deep sequencing results of gene edited of CCR5 in bamboo leaves. Portions of sequences in red, blue, and grey indicated the target sites, PAM, and insertions, respectively. The red dashes indicated the deleted nucleotides. **Fig. S11. **Deep sequencing results of CCR4 by the gRNA of CCR5 in bamboo leaves. Portions of sequences in red, blue, and grey indicated the target sites, PAM, and insertions, respectively. The red dashes indicated the deleted nucleotides. **Fig. S12. **Deep sequencing results of CCR5 by the gRNA of CCR4 in bamboo leaves. Portions of sequences in red, blue, and grey indicated the target sites, PAM, and insertions, respectively. The red dashes indicated the deleted nucleotides. **Fig. S13. **The measurement of flavonoid and lignin content in the bamboo leaves. **a**, Total flavonoid content in the infected leaves with lower NPQ values. **b,** Lignin content in the infected leaves with lower NPQ values. The green and red filled columns represented the decrease and increase of flavonoid content compared with the control, respectively. Data are means ± AVEDEV (n=3). * and ** indicated significant differences at *p*<0.05 and *p*<0.01 by ANOVA, respectively. Exact *p* values from CCR1 to CCR5 were as follows: **a** 0.0945, 0.0358, 0.0242, 0.0069, 0.2445, **b** 0.853, 0.9798, 0.0264, 0.026, 0.9718.

**Additional file 2: Table S1**. The percentage of seedlings accumulated betalain after being infected by Agrobacterium strains harboring 35S:RUBY construct.

**Additional file 3: Table S2**. The sequence information of primers.

## Data Availability

The data that support the findings of this study are available from the corresponding author upon request, and detailed information of the primer and gRNA sequences used in this study is provided in the Additional file documents.
