# Supplementary material for: A new biotechnology for in-planta gene editing and its application in promoting flavonoid biosynthesis in bamboo leaves
Source: Plant Methods. 2023 Mar 2;19:20. doi: 10.1186/s13007-023-00993-4 (PMC9979463; doi:10.1186/s13007-023-00993-4)

**Additional file**

**Fig. S1.** The leaf phenotype of *Phyllostachys edulis* seedlings with betalain accumulation. The *Agrobacterium tumefaciens* strains of AGL1, LBA4404, EHA105, and GV3101 harboring *35S*::*RUBY* construct were used to infiltrate by vacuum respectively. The red triangles indicated the positions of the wounds.


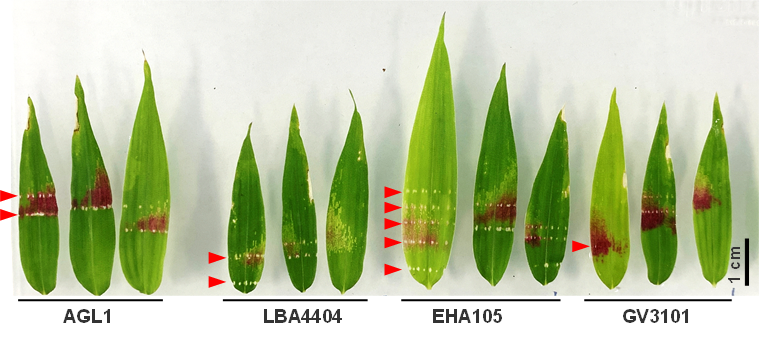


**Fig. S2.** The shoot phenotype of *Phyllostachys aureosulcata* '*Spectabilis*' with betalain accumulation after infection for 15 days. **a,** Shoots injected with *Agrobacterium*-free suspension. **b,** Shoots injected with the buffer solution with *Agrobacterium* suspension harboring *35S*::*RUBY* construct. Black box, Magnified image.


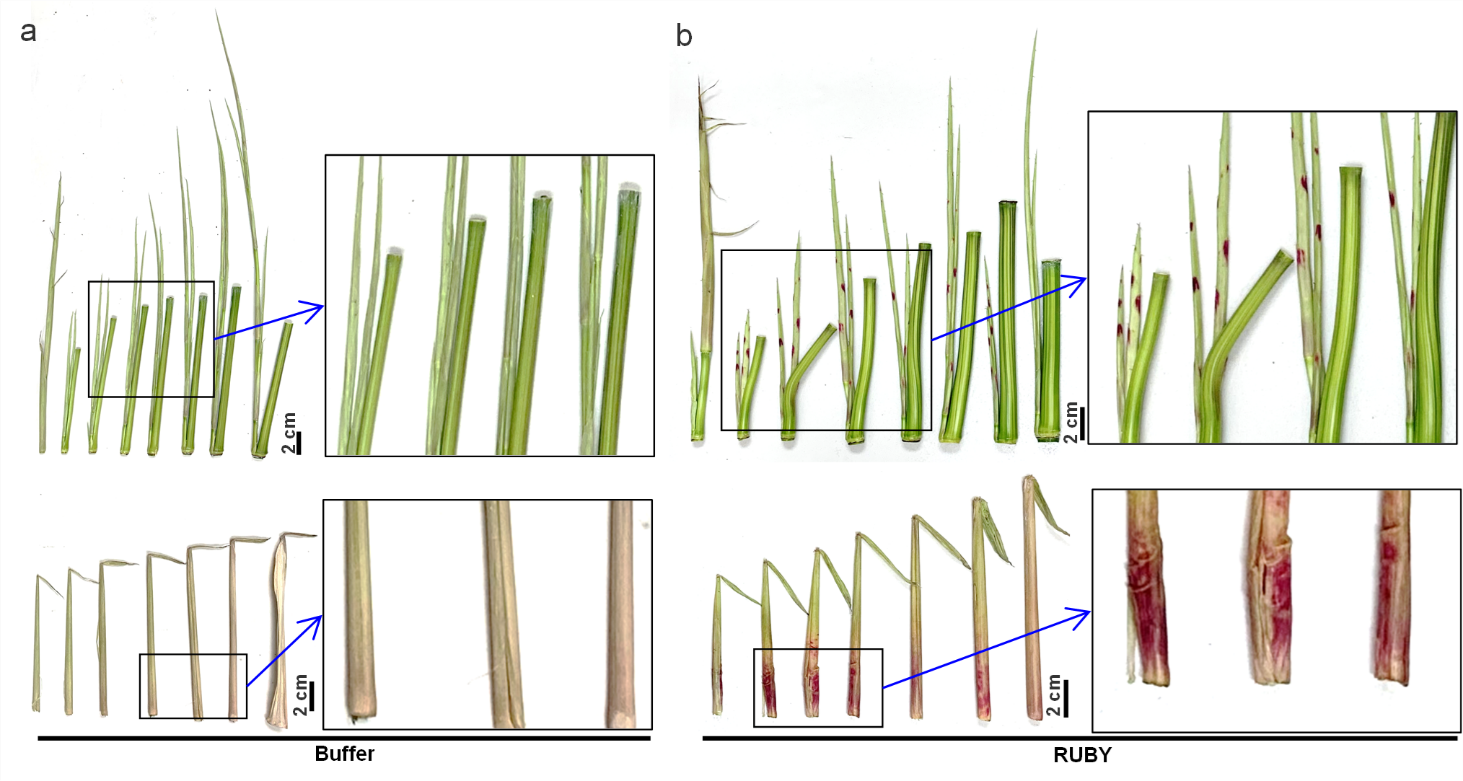


**Fig. S3.** The shoot phenotype of *Phyllostachys aureosulcata* '*Aureocarlis*' with betalain accumulation after infection for 15 days. **a,** Shoots injected with *Agrobacterium*-free suspension. **b,** Shoots injected with the buffer solution with *Agrobacterium* suspension harboring *35S*::*RUBY* construct. Black box, Magnified image.


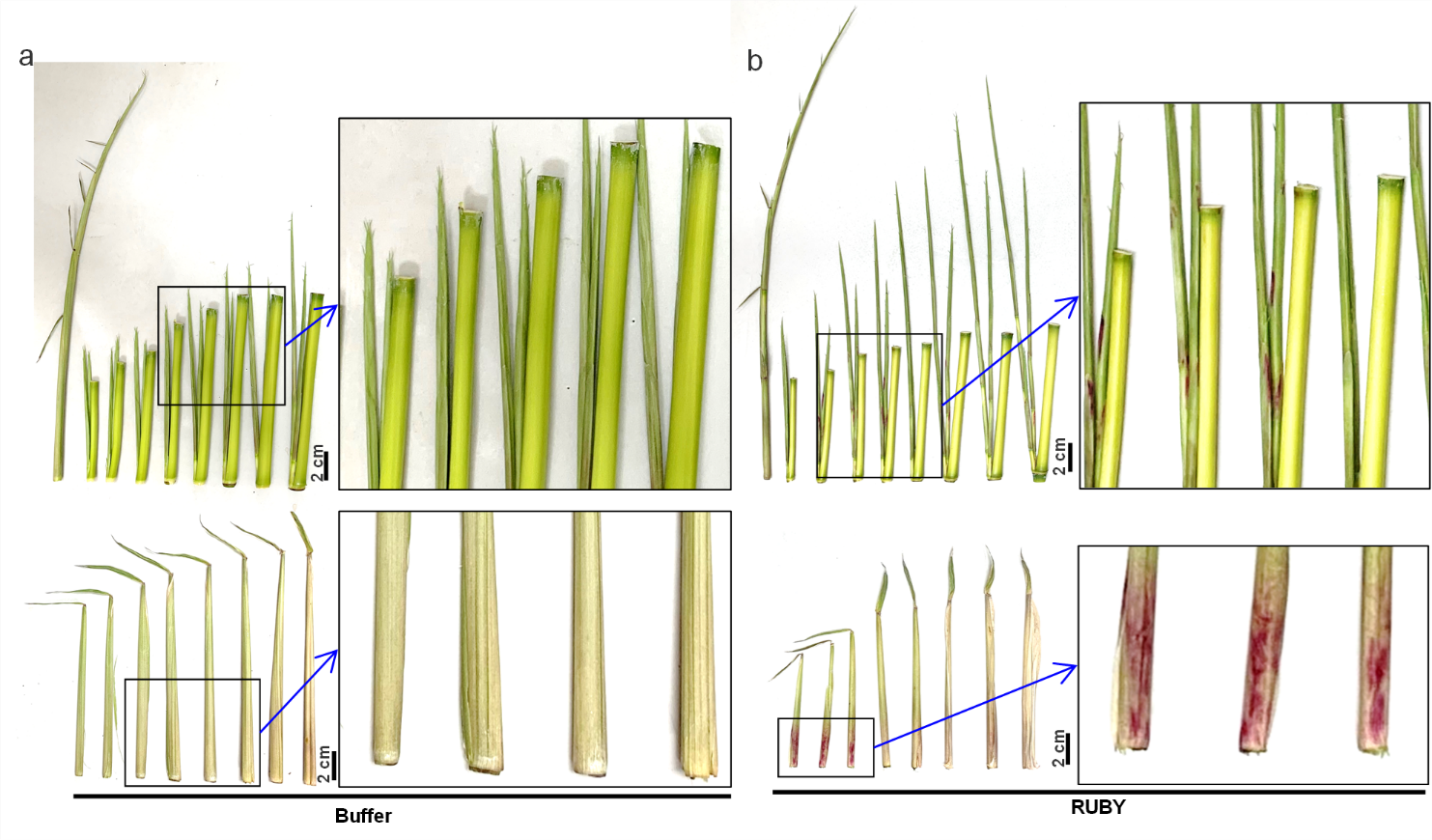


**Fig. S4.** *RUBY* reporter gene detected in the red leaf areas with betalain accumulation using PCR. Lane1, the non-infected leaves as the negative control; Lane2 and 3, the betalain accumulated leaves after *Agrobacterium* infection for three days and 30 days respectively; Lane4, the *35S*::*RUBY* plasmid as positive control. *PeActin* (GenBank Accession No. GU434145) was used as endogenous control under the same PCR conditions.


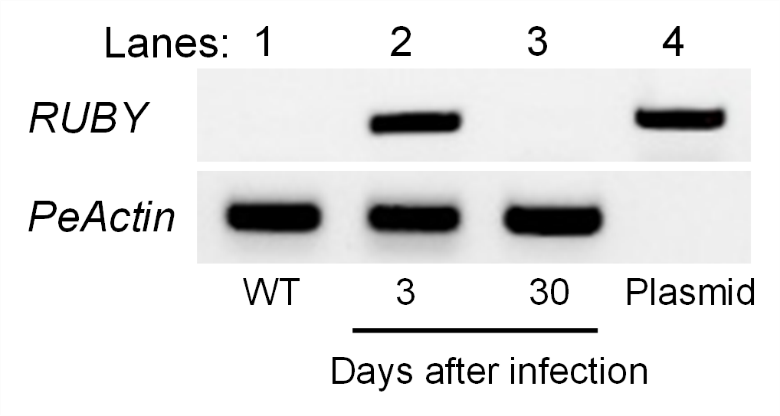


**Fig. S5.** The detection of gene-edited target1 of *PeVDE* in bamboo leaves. **a,** Results of PCR of genomic DNA obtained from wild-type (WT) non-infected and infected leaves using gRNA-1 after infection for 5 or 10 days. + and - represent PCR products with or without *Xba*I digestion. The different bands were indicated with red arrows. **b–c,** Deep sequencing results of wide-type (**b**) and mutated *PeVDE* clones edited using gRNA-1 (**c**). Portions of sequences in red, blue, and grey indicated the target sites, PAM, and insertions, respectively. The red dashes indicated the deleted nucleotides.


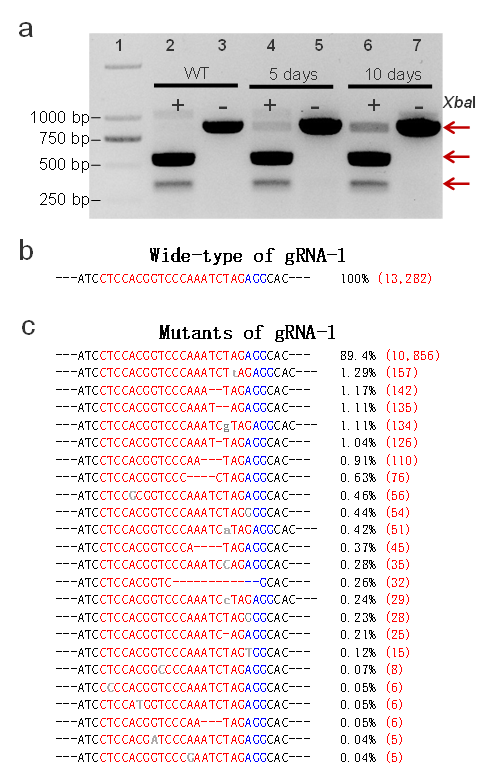


**Fig. S6.** Deep sequencing results of gene edited of CCR1 in bamboo leaves. Portions of sequences in red, blue, and grey indicated the target sites, PAM, and insertions, respectively. The red dashes indicated the deleted nucleotides.

**
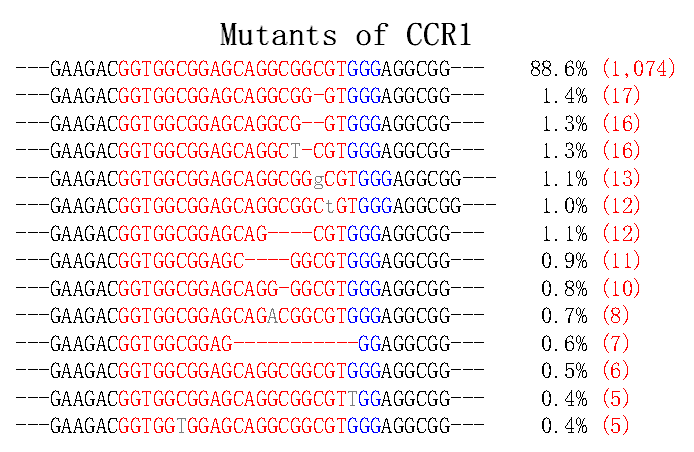
**

**Fig. S7.** Deep sequencing results of gene edited of CCR2 in bamboo leaves. Portions of sequences in red, blue, and grey indicated the target sites, PAM, and insertions, respectively. The red dashes indicated the deleted nucleotides.

**
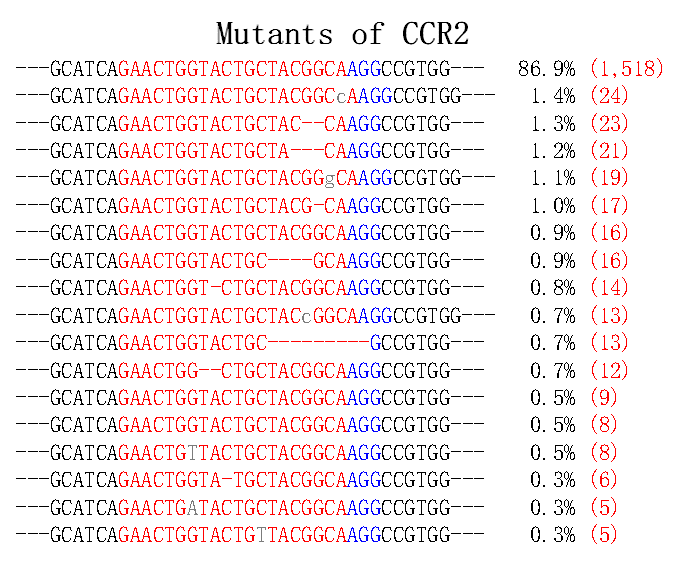
**

**Fig. S8.** Deep sequencing results of gene edited of CCR3 in bamboo leaves. Portions of sequences in red, blue, and grey indicated the target sites, PAM, and insertions, respectively. The red dashes indicated the deleted nucleotides.

**
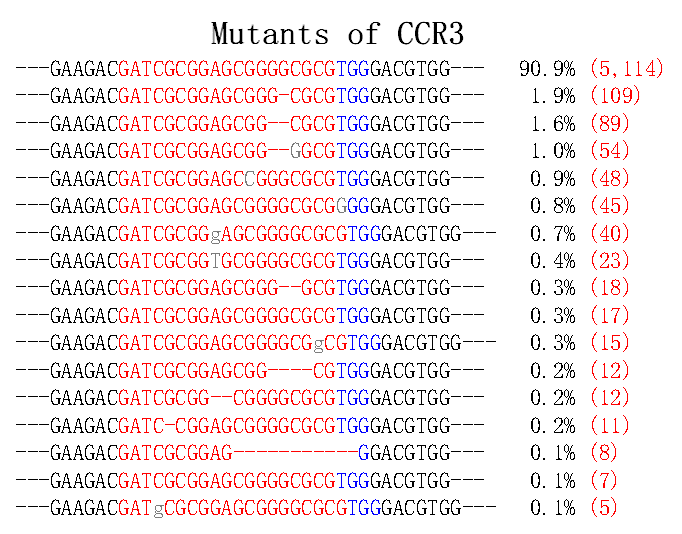
**

**Fig. S9.** Deep sequencing results of gene edited of CCR4 in bamboo leaves. Portions of sequences in red, blue, and grey indicated the target sites, PAM, and insertions, respectively. The red dashes indicated the deleted nucleotides.

**
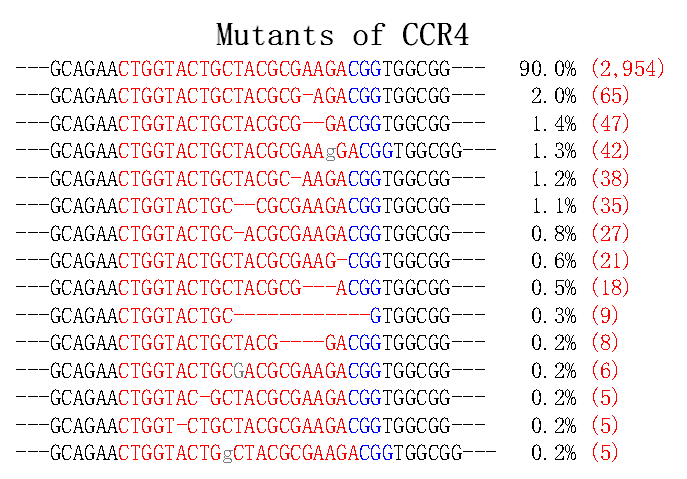
**

**Fig. S10.** Deep sequencing results of gene edited of CCR5 in bamboo leaves. Portions of sequences in red, blue, and grey indicated the target sites, PAM, and insertions, respectively. The red dashes indicated the deleted nucleotides.


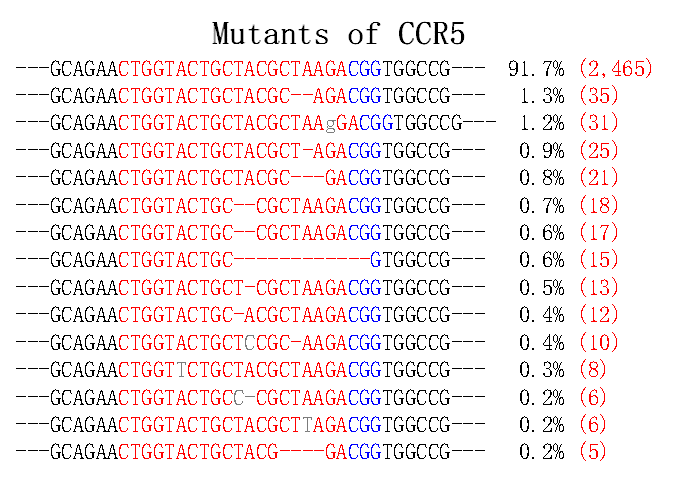


**Fig. S11.** Deep sequencing results of CCR4 by the gRNA of CCR5 in bamboo leaves. Portions of sequences in red, blue, and grey indicated the target sites, PAM, and insertions, respectively. The red dashes indicated the deleted nucleotides.


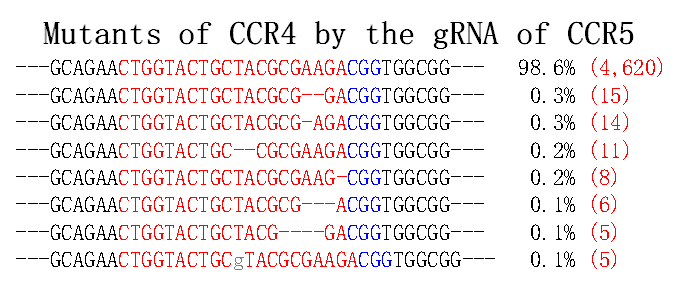


**Fig. S12.** Deep sequencing results of CCR5 by the gRNA of CCR4 in bamboo leaves. Portions of sequences in red, blue, and grey indicated the target sites, PAM, and insertions, respectively. The red dashes indicated the deleted nucleotides.


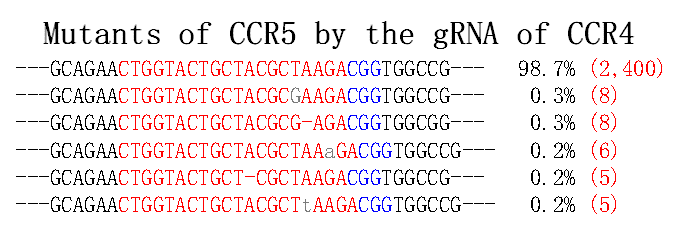


**Fig. S13.** The measurement of flavonoid and lignin content in the bamboo leaves. **a,** Total flavonoid content in the infected leaves with lower NPQ values. **b,** Lignin content in the infected leaves with lower NPQ values. The green and red filled columns represented the decrease and increase of flavonoid content compared with the control, respectively. Data are means ± AVEDEV (n=3). * and ** indicated significant differences at *p*<0.05 and *p*<0.01 by ANOVA, respectively. Exact *p* values from CCR1 to CCR5 were as follows: **a** 0.0945, 0.0358, 0.0242, 0.0069, 0.2445, **b** 0.853, 0.9798, 0.0264, 0.026, 0.9718.


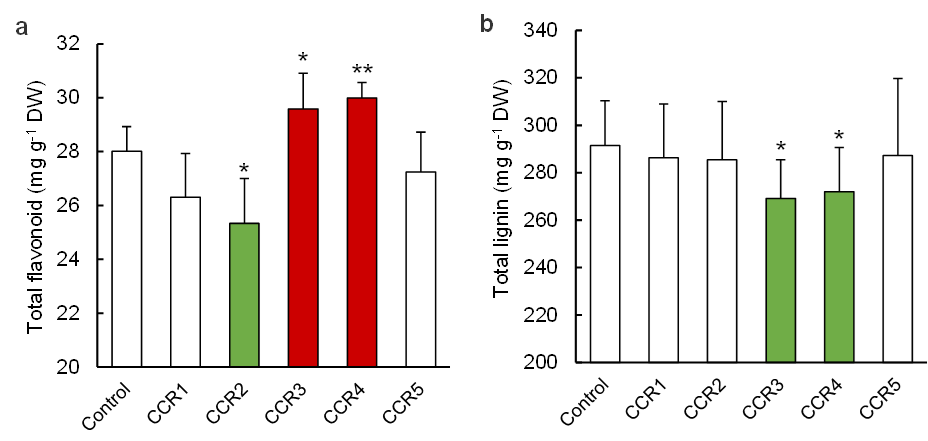

Supplement: Supplementary file 1 — Additional file 1: Fig. S1. The leaf phenotype of Phyllostachys edulis seedlings with betalain accumulation. The Agrobacterium tumefaciens strains of AGL1, LBA4404, EHA105, and GV3101 harboring 35S::RUBY construct were used to infiltrate by vacuum respectively. The red triangles indicated the positions of the wounds. Fig. S2. The shoot phenotype of Phyllostachys aureosulcata Spectabilis’ with betalain accumulation after infection for 15 days. a, Shoots injected with Agrobacterium-free suspension. b, Shoots injected with the buffer solution with Agrobacterium suspension harboring 35S:RUBY construct. Black box, Magnified image. Fig. S3. The shoot phenotype of Phyllostachys aureosulcata 'Aureocarlis' with betalain accumulation after infection for 15 days. a, Shoots injected with Agrobacterium-free suspension. b, Shoots injected with the buffer solution with Agrobacterium suspension harboring 35S:RUBY construct. Black box, Magnified image. Fig. S4. RUBY reporter gene detected in the red leaf areas with betalain accumulation using PCR. Lane1, the non-infected leaves as the negative control; Lane2 and 3, the betalain accumulated leaves after Agrobacterium infection for three days and 30 days respectively; Lane4, the 35S::RUBY plasmid as positive control. PeActin (GenBank Accession No. GU434145) was used as endogenous control under the same PCR conditions. Fig. S5. The detection of gene-edited target1 of PeVDE in bamboo leaves. a, Results of PCR of genomic DNA obtained from wild-type (WT) non-infected and infected leaves using gRNA-1 after infection for 5 or 10 days. + and - represent PCR products with or without XbaI digestion. The different bands were indicated with red arrows. b–c, Deep sequencing results of wide-type (b) and mutated PeVDE clones edited using gRNA-1 (c). Portions of sequences in red, blue, and grey indicated the target sites, PAM, and insertions, respectively. The red dashes indicated the deleted nucleotides. Fig. S6. Deep sequencing results of gene edi [file 13007_2023_993_MOESM1_ESM.docx]
